# Age-related degradation of behavioral and network features of *Aplysia* escape locomotion

**DOI:** 10.3389/fnagi.2026.1807660

**Published:** 2026-04-10

**Authors:** Viral K. Mistry, Daniel Martinez, William N. Frost

**Affiliations:** 1 Stanson-Toshok Center for Brain Function and Repair, Rosalind Franklin University of Medicine and Science, North Chicago, IL, United States; 2 College of Pharmacy, Rosalind Franklin University of Medicine and Science, North Chicago, IL, United States

**Keywords:** aging, *Aplysia*, escape locomotion, motor programs, VSD imaging

## Abstract

**Introduction:**

*Aplysia californica* has been a useful model system for studies of the neural basis of behavior, learning, and aging. While the latter topic has been explored with respect to several of its simple reflex behaviors, this study represents the first examination of how one of Aplysia’s more complex behaviors, escape locomotion, is affected in animals nearing the end of their natural lifespan.

**Methods:**

Middle-aged (5-7 mo) and elderly (12-13 mo) *Aplysia* were subjected to an experimental or control stimulus protocol (*n* = 16 each group). Head reach latency, gallop share, and cycle number were calculated for each animal. Voltage-sensitive dye (VSD) imaging was performed on the isolated brains of middle-aged or elderly animals (*n* = 6 each group) under an experimental or control fictive locomotion stimulus protocol. Onset latency, cycle number, and average spikes/cell were calculated for each recording. VSD imaging was also done on middle-aged and elderly brains at rest (*n* = 7 each group), and average # of spikes/cell as well as burst duration, intraburst frequency, and cycle/min were calculated for each recording.

**Results:**

Elderly *Aplysia* showed greatly reduced gallop response, and a loss in head reach latency sensitization in both intact animals and isolated brains. Onset latency sensitization was also more transient in middle-aged isolated brains than in middle-aged intact animals. Repeated stimulation in middle-aged isolated brains increased average spikes/cell, but elderly brains saw decreased average spikes/cell under the same protocol. Resting state activity was also weaker and slower in elderly isolated brains.

**Discussion:**

This study provides the first evidence of how aging changes behavioral and network parameters of *Aplysia* escape locomotion, including a potential early indicator of age-related dysfunction present in middle-aged brains that has yet to emerge in the behavior. This supports future work investigating compensatory mechanisms and opportunities for future therapeutic interventions.

## Introduction

Aging results in progressive decline in normal physiological function across species (López-Otín et al., 2013; López-Otín et al., 2023). In the nervous system, aging causes cellular and molecular changes that result in impaired behavioral function and reduced memory formation. This includes reduced synaptic plasticity, reduced neurotransmitter expression, the loss of neurons, and reduced circuit activity (Yankner et al., 2008). The comparative simplicity of invertebrate networks and their typically shorter lifespan creates an opportunity for their use to study the neural correlates of age-related behavioral and cognitive decline (Biga et al., 2025; Yeoman et al., 2012). Investigations into *C. elegans* and *Drosophila melanogaster*, for instance, have taken advantage of these species’ simpler physiology, nervous systems, and behaviors to provide new insights into the cellular and molecular mechanisms of aging (Son et al., 2019; Liu et al., 2013; Olsen et al., 2006; Overman et al., 2022; He and Jasper, 2014; Iliadi et al., 2012). However, there remains a need for simple models that can address the larger network-level components of behavioral and cognitive aging and test potential interventions for rescuing decline.

*Aplysia californica* is an invertebrate gastropod mollusk with a repertoire of behaviors that have been the basis of many foundational investigations into the neural basis of behavior and learning (Kandel, 2001; Bédécarrats and Nargeot, 2020). *Aplysia* also undergoes progressive behavioral and neurophysiological decline as it ages (Kempsell and Fieber, 2014; Bailey et al., 1983), and can be acquired at any specified age from the National Resource for *Aplysia*, an NIH-supported facility where they are raised from eggs to adulthood under controlled conditions (Gerdes and Fieber, 2006). Aged *Aplysia* display cellular, molecular, and behavioral changes that have made them a valuable model system for studying age-related behavioral and cognitive decline (Kempsell and Fieber, 2015; Kempsell and Fieber, 2016; Kron and Fieber, 2022; Badal et al., 2024; Rattan and Peretz, 1981; Fieber et al., 2010; Hallahan et al., 1992; Peretz and Srivatsan, 1992). However, these previous studies have largely focused on simple reflexes, such as those mediating tail and gill-siphon withdrawals. *Aplysia* also possesses more complex behaviors, such as escape locomotion, which has two phases - an initial fast “gallop” phase which transitions into a slower “crawl” phase (Flinn et al., 2001). The behavior arises from a low-dimensional spiral attractor network state that drives neuronal ensembles spatially and functionally clustered within the pedal ganglia (Bruno et al., 2015; Bruno et al., 2017). A partially defined central pattern generator produces this behavior by driving a large population of efferent neurons to rhythmically burst for many minutes, producing a repeating wave of head-to-tail muscle contraction that propels the animal forward (Jahan-Parwar and Fredman, 1979; Fredman and Jahan-Parwar, 1980; Wang et al., 2023). The motor network is activated by serotonin, with key modulatory neurons located in the pedal and cerebral ganglia (Wang et al., 2023; Fredman and Jahan-Parwar, 1983; Jing et al., 2008). Exploration into learning in this behavior, however, has been limited (Stopfer and Carew, 1988; Walters, 1980), and no prior study has explored how said learning is affected by aging. This presents an opportunity to investigate how aging affects both motor and cognitive function in a more complex behavior than those simple reflexes within the same simple tractable nervous system. Working out concrete cellular and network mechanisms underlying motor and cognitive aging in this behavioral network may generate new insights into aging at a network level across species.

In this study, we demonstrate for the first time that elderly *Aplysia* display behavioral and cognitive aging of specific parameters of their escape locomotion behavior and network. Aged *Aplysia* lose the ability to behaviorally sensitize locomotion onset and show significant decline in their galloping capacity. Isolated brain preparations also display similar deficits as seen in the intact behavior, demonstrating a CNS origin of some of these deficits. We also observe deficits in isolated brains not yet seen in intact behavior, a potential early signature of cognitive decline in the brain that presents future opportunities to explore potential therapeutic interventions. Elderly *Aplysia* brains also responded less vigorously to an initial stimulus than middle-aged adult brains. With repeated stimulation, we observed inverse effects with respect to age – successive stimuli produced increased responsiveness during the motor program in middle-aged brains, but produced decreased responsiveness in old brains. We also show that the resting state activity of elderly *Aplysia*’s motor network is reduced with age, suggesting the presence of a broader age-related network degeneration.

These findings demonstrate that aging has distinct and heterogeneous effects on the *Aplysia* escape locomotion network and establish this system as a model for further investigations into the cellular, molecular, and network dynamics of behavioral and cognitive aging. This study will also inform future work to investigate methods of mitigating or rescuing this decline.

## Methods

*Preparation – Aplysia californica* of two ages were obtained from the National Resource for *Aplysia* at the University of Miami’s Rosenstiel School of Marine, Atmospheric, and Earth Science (Coral Gables, FL). Animal hatch date was provided with each shipment. The median life expectancy of these *Aplysia* is around 13–14 months (Gerdes and Fieber, 2006). For this study, middle-aged adult *Aplysia* were defined as 5–7 mo, and weighed between 20 and 40 g. Elderly adult *Aplysia* were defined as 12–13 mo and weighed between 100 and 250 g. Animals were maintained in chilled (13.5 °C–16.5 °C) recirculating artificial seawater (ASW) systems prior to experiments. The ASW was made using Instant Ocean mix (Spectrum Brands LLC). Experiments involving isolated brains were performed in filtered ASW.

*Behavioral experiments - Aplysia* were weighed and then placed alone into the top tier of a 5 ft. long plexiglass recirculating artificial seawater tank. One hour after being moved into the testing region, locomotion was elicited by applying 1 mL of 5 M NaCl to the tail of the animal. Locomotion was filmed continuously from before until 2 min after an aversive salt stimulus was applied to the tail, using a digital camcorder positioned to view the lateral profile of the entire animal. Videos were analyzed and hand-scored for behavioral features using ShotCut (Meltytech, LLC). Head reach latency was measured as the time between the beginning of the application of the NaCl solution to the maximum of the first head extension. Gallops and crawls were distinguished as previously defined in (Flinn et al., 2001; Hill et al., 2020): gallop cycles involve an arched head reach followed by a simultaneous mid-body & tail pull, while crawl cycles involve a head-to-tail rolling wave of muscle contraction that moved the animal forward without the distinctive midbody arch of the gallop. A plexiglass wall was used in an effort to guide the animals down the length of the tank in such a manner that their side profile remained in easy view from the camera. However, as the animals had a tendency to occasionally crawl up the side of the tank or at an angle away from the camera, distance traveled could not be consistently measured as they were not always locomoting in a straight line.

*Optical recordings* - In experiments where we sought to characterize the efferent locomotion network, we optically recorded voltage activity using a fast voltage-sensitive dye to record the action potentials of rhythmically active gallop/crawl neurons in the pedal ganglion, as in (Bruno et al., 2015; Bruno et al., 2017; Hill et al., 2020). *Aplysia’s* central ganglia, consisting of the cerebral ganglion connected to the left & right pleural and pedal ganglia, were dissected and pinned to the bottom of a Sylgard (Dow Corning) lined Petri dish containing filtered ASW. The ASW was recirculated through the dish via a peristaltic pump (Model 720, Instech Laboratories). During the entire experiment, the temperature in the chamber was maintained between 14.0 °C and 15.0 °C by having the filtered ASW pass through a feedback-controlled in-line Peltier cooling system (Model SC-20, Warner Instruments) before it entered the chamber. The Peltier’s heat was carried away via a separate recirculating liquid cooling system (EX2-1055, Koolance). Temperature in the chamber was monitored with a BAT-12 thermometer fitted with an IT-18 microprobe (Physitemp Instruments). Pedal ganglion neurons were stained by periodically applying pressure to a PE tube filled with 0.2 mg/mL RH-155 (Toronto Research Chemicals, Toronto, CA), pressed against the surface of the ganglion for 1 h. Following staining, the pedal ganglion was transferred and pinned to a Slygard-lined perfusion chamber used for optical recording, where the PdN9, a tail nerve, contralateral to the stained pedal ganglion was pulled into a suction electrode. To improve visualization, two small pieces of silicone (Mack’s, McKeon Products) were placed in the chamber on opposite sides of the stained ganglion, allowing for a glass coverslip fragment to be pressed down and held in place to flatten the surface of the stained pedal ganglion (as described in (Hill et al., 2020)). The preparation was trans-illuminated by a 735 nm LED (Thor Labs), and the light was collected by a 10× 0.6 NA water-immersion objective lens (Olympus) and passed through a phototube to reach either the photodiode array or a parfocal camera (Optronics). The preparation rested on the optical imaging rig for 60 min following nerve suction before acquisitions began. Fictive escape locomotion motor programs were elicited via stimulation of the contralateral PdN9 (8 V, 20 Hz, 2.5 s stimulus train, 5 ms pulses). Voltage activity was captured using a RedShirtImaging PDA-III photodiode array consisting of 464 photodiodes sampled at 1600 Hz. Before sampling, all traces were AC-coupled in hardware to zero the baseline of the traces (2-s time constant) and then amplified 100x by the PDA-III. After acquisition, optical data were band-pass filtered in the Neuroplex software (Butterworth, 5 Hz high pass, 100 Hz low pass) and saved as text files. Independent component analysis (ICA) was run on the optical data in MATLAB (Mathworks), as made available in (Hill et al., 2010), to obtain action potentials from individual neurons. Following ICA, the components had a manual threshold applied to convert the action potential spike trains into binary spike times for further analysis of neuronal activity.

*Analysis of spike trains generated via optical recordings* - Spike times were calculated from the binary spike train files generated from the optical recordings. Neural correlates of behavior (motor program onset latency and cycle number) were calculated using a custom MATLAB script to determine the time from stimulus onset to the termination of the first rhythmic burst (onset latency) as well as the number of rhythmic bursts within the first 2 minutes from stimulus onset (cycle number). Spike times were used to generate spike frequency in 1-s time bins for each neuronal trace and averaged for each recording to generate average spike frequency. An unsupervised consensus clustering algorithm described in (Bruno et al., 2015) was used to determine functional ensembles in the motor program and to identify candidate rhythmic ensembles in recordings of spontaneous activity. Custom MATLAB script was used to determine rhythmicity within an averaged ensemble: Welch’s method was used to calculate power spectral density and spectral peaks with adaptive prominence were identified. Peak power ratio (max peak power divided by mean power), coefficient of variation of the spectrum, and spectral entropy were used in the following equation to generate a “rhythmicity score”:


$$\begin{array}{l} \text{Rhythmicity Score}=\text{Peak Power Ratio}*\\ \;\;\;\;\;\;\;\;\;\;\mathrm{CV}\;\text{of Spectrum}*(1-\text{Spectral Entropy}) \end{array}$$

A positive rhythmicity score indicated a rhythmic cluster. If a cluster was determined to be rhythmic, the burst duration, intraburst frequency, and cycle frequency for every burst performed by the cluster during the recording were then calculated. From these values, the mean burst duration, intraburst frequency, and cycle frequency were then calculated for all the rhythmic clusters within a single recording.

## Results

### Elderly *Aplysia* cannot sensitize onset latency and lose galloping capacity

**Figure 1:**
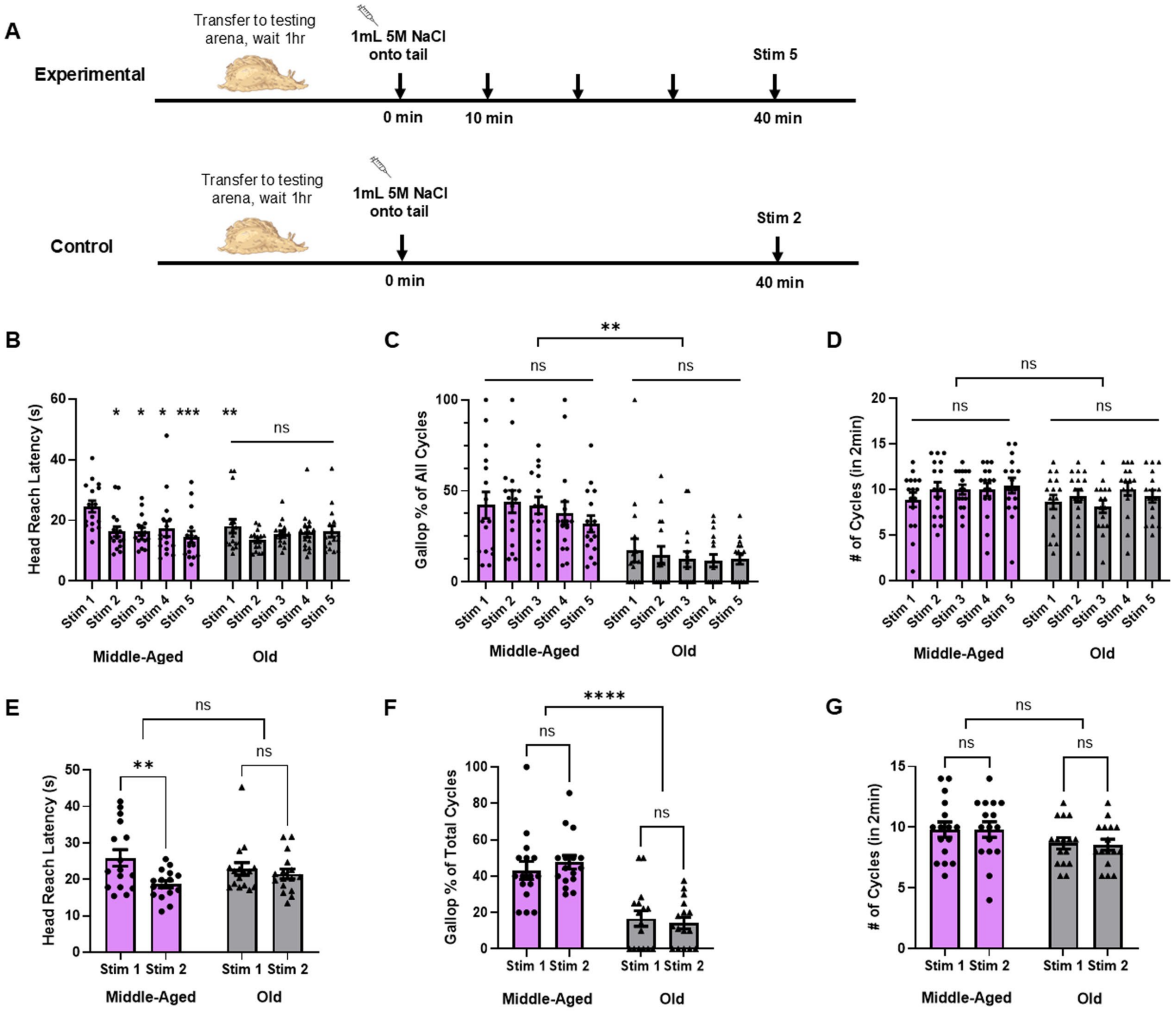
Aging causes decline in specific parameters of escape locomotion behavior. **(A)** Behavioral protocol used in middle-aged and old *Aplysia*, with experimental group (*n* = 16 for middle-aged and old) receiving 5 stimuli at 10 min ISI and control group (*n* = 16 for middle-aged and old) receiving 2 stimuli at 40 min ISI (equivalent time as experimental). **(B)** Head reach latency significantly quickened after an initial stimulus in middle-aged *Aplysia* and remained quickened with successive stimuli (* for *p* < 0.05, *** for *p* < 0.0001). Head reach latency to stim 1 in middle-aged *Aplysia* is also significantly slower than latency to stim 1 in old *Aplysia*. **(C)** Gallop share of all locomotion cycles was unchanged within middle-aged and old groups, but was significantly elevated in middle-aged versus old (** for *p* < 0.01). **(D)** No change in cycle number produced within 2 min in either middle-aged or old *Aplysia*. **(E)** Head reach latency is significantly quickened in middle-aged *Aplysia* in control condition, demonstrating persistent sensitization. **(F)** Gallop share in control condition demonstrates again only age effect, not effect from stimulation. **(G)** Cycle number in control condition shows no effect from stimulation or between groups.

To determine the effects of aging on *Aplysia’s* escape locomotion behavior and capacity for learning, we developed a same-site behavioral non-associative learning protocol for this behavior that we then applied to both middle-aged adult (5–7 mo) and elderly adult *Aplysia* (12–13 mo). In this behavioral paradigm, we applied a repeated same-site aversive salt stimulus (1 mL 5 M NaCl) to the animal’s tail. In the experimental group (*n* = 16 for both middle-aged and old animals), we provided 5 stimuli 10 min apart. In the control group (*n* = 16 for both middle-aged and old animals), we provided 2 stimuli 40 min apart – the equivalent amount of time as the experimental group (Figure 1A). We then analyzed various parameters of the behavior to identify potential behavioral features of either sensitization (increased response) or habituation (decreased response). We chose three behavioral parameters (head reach latency, cycle number within 2 min of the stimulus, and the percentage of locomotion cycles that are gallops) that were easily quantifiable and represented different features of locomotion to see if age-related decline differentially affected the overall behavior. Head reach latency represented locomotion onset, cycle # represented duration, and the share of cycles that were gallop vs. crawl represented intensity.

In the experimental group, a RM two-way ANOVA did not find significance in the interaction between age and repeated stimulation (*F*(4, 75) = 1.67, *p* = 0.17) and did find a significant effect of repeated stimulation on head reach latency (*F*(4, 75) = 3.859, *p* = 0.007) (Figure 1B). Multiple comparisons with Tukey’s post-hoc tests revealed a quickening of head reach latency from stim 1 to stim 2 in middle-aged *Aplysia* (stim 1: 24.6 ± 1.9 s vs. stim 2: 16.3 ± 1.6 s, *p* = 0.01). This quickening of head reach latency persisted with each follow-up stimulus for the middle-aged age group (stim 3: 16.3 ± 1.3 s, *p* = 0.01; stim 4: 16.6 ± 2.6 s, *p* = 0.03; stim 5: 14.6 ± 1.9 s, *p* = 0.0009). By comparison, there was no change in head reach latency from stim 1 to stim 2 in old *Aplysia* (stim 1: 18.1 ± 2.3 s vs. stim 2: 13.5 ± 0.8 s, *p* = 0.37), or in any follow up stimuli. Head reach latency to the first stimulus was also significantly different between middle-aged and old *Aplysia* in the experimental group (*p* = 0.008). In the control group, a RM two-way ANOVA did find significance in the interaction between the stimuli and age (*F*(1, 15) = 5.824, *p* = 0.02) and also revealed a significant effect from the stimuli (*F*(1, 15) = 9.911, *p* = 0.007) (Figure 1E). Tukey’s post-hoc tests also revealed a significant quickening of head reach latency in the control group from stim 1 to stim 2 in middle-aged *Aplysia* (stim 1: 25.9 ± 2.3 s vs. stim 2: 18.7 ± 0.9 s, *p* = 0.004), but no change in head reach latency from stim 1 to stim 2 in old *Aplysia* (stim 1: 22.9 ± 1.7 s vs. stim 2: 21.5 ± 1.3 s, *p* = 0.85). These results reveal an identified cognitive feature of *Aplysia*’s locomotion behavior – sensitization of head reach latency – that is lost with aging. This behavioral sensitization in middle-aged animals persisted with repeated stimuli and as shown by the control group, lasted after a single trial for at least 40 min.

The experimental group observed no change in gallop share over repeated stimulation within either the middle-aged or old groups (*F*(4, 75) = 0.77, *p* = 0.55), nor was the interaction between repeated stimulation and age significant (*F*(4, 75) = 0.29, *p* = 0.88) (Figure 1C). There was, however, a significant difference between the middle-aged and old groups in gallop share (*F*(1, 75) = 57.91, *p* < 0.0001, mean of middle-aged: 39.6% vs. mean of old: 13.8%). The control group displayed the same phenomenon: no significance in the interaction between stimulation and age (*F*(1,15) = 2.008, *p =* 0.18) and no effect from stimulation within either age group (*F*(1, 15) = 0.1055, *p* = 0.75), but a difference between the age groups (F(1, 15) = 37.04, *p* < 0.0001, mean of middle-aged: 45.4% vs. mean of old: 15.5%) (Figure 1F). Therefore, this stimulus protocol did not produce learning-induced changes in galloping in middle-aged or old animals and instead revealed an age-based behavioral loss in galloping that could not be rescued by repeated stimulation.

Meanwhile, the number of cycles performed within the first 2 min following the stimulus in the experimental group was unchanged by repeated stimulation (*F*(4, 75) = 1.021, *p* = 0.40) or by age (*F*(1, 75) = 3.160, *p* = 0.08, mean of middle-aged: 9.9 cycles vs. mean of old: 9.1 cycles) and showed no significance in the interaction of stimulation and age (*F*(4, 75) = 0.6023, *p* = 0.66) (Figure 1D). In the control group we also found cycle number did not show significant interactions between stimulus and age (*F*(1, 15) = 0.016, *p* = 0.90), and did not change from follow-up stimulation (*F*(1, 15) = 0.0134, *p* = 0.91) or between age groups (F(1, 15) = 3.871, *p* = 0.07, mean of middle-aged: 9.8 cycles vs. mean of old: 8.6 cycles) (Figure 1G). Thus, this behavioral parameter did not develop any learning induced changes via this behavioral protocol in either age group, and did not display any significant age-related decline.

Here, we demonstrated that aging is associated with a loss of sensitization learning in a specific parameter of locomotion – head reach latency. A different parameter, gallop share, was unchanged by learning but was diminished with aging. Yet another parameter, cycle number, was unchanged by both learning and aging. Aging thus degrades both motor and cognitive abilities in *Aplysia’s* locomotion behavior.

### Isolated brains from old animals also cannot sensitize onset latency and show reduced cycling in the fictive motor program

**Figure 2:**
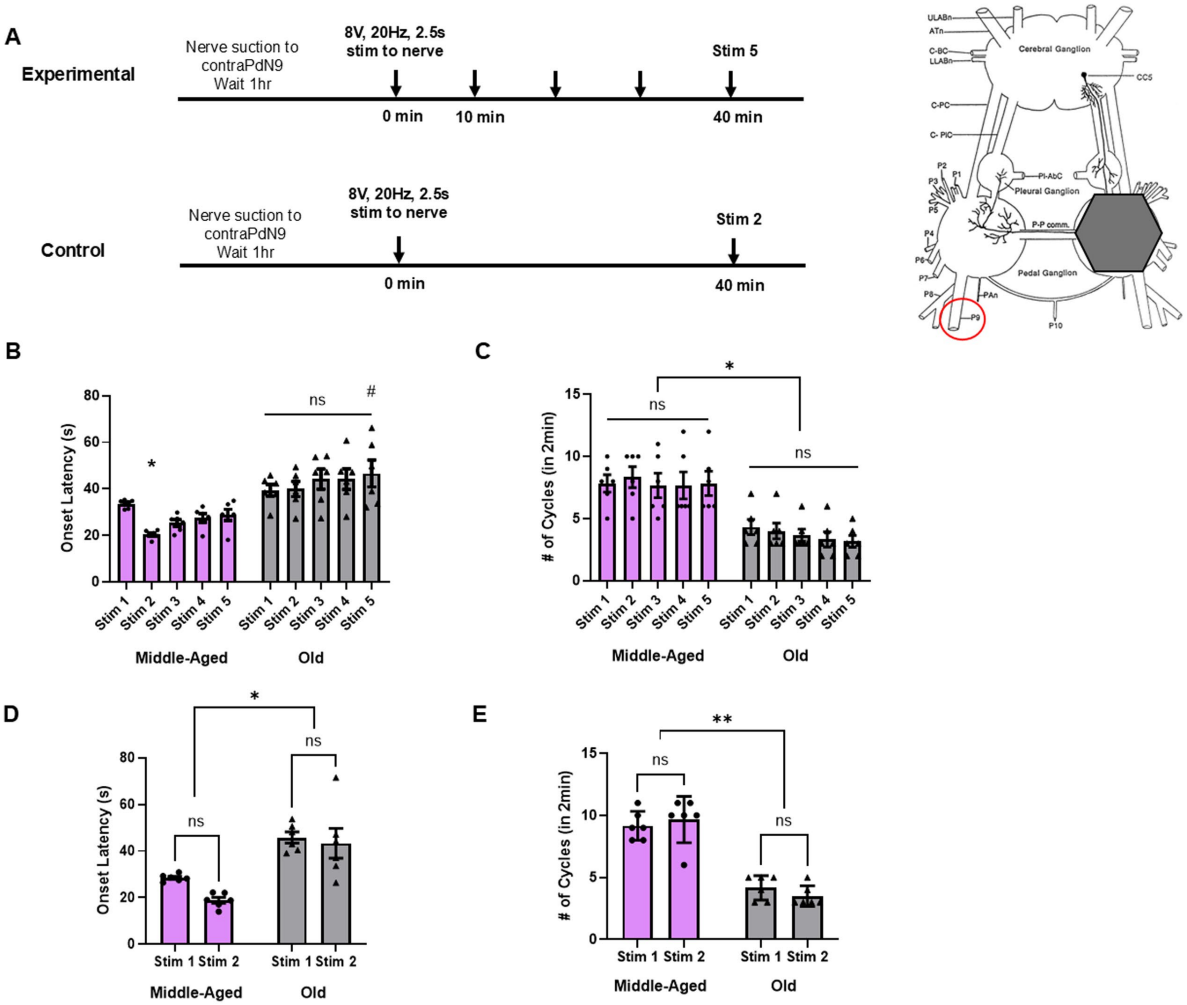
Aging also produces deficits in fictive locomotion in isolated brains. **(A)** *Left, p*rotocol for stimulation in isolated brains, with the experimental group (*n* = 6 for middle-aged and old) receiving 5 stimuli to PdN9 at 10 min ISI, and control group (*n* = 6 for middle-aged and old) 2 stimuli at 40 min ISI (equivalent time as experimental). *Right*, schematic of isolated central ganglia of *Aplysia*, as adapted from (Xin et al., 1996). Grey hexagon indicates region where VSD imaging is taking place (either left or right dorsal pedal ganglion). Red circle indicates the contralateral nerve stimulated during VSD imaging, Pedal Nerve 9. **(B)** Onset latency only shows quickening from stim 1 to stim 2 in middle-aged brains (* for *p* < 0.05). Old brains show no change in onset latency with repeated stimulation. Onset latency to stim 5 in old brains is significantly slower than stim 5 in middle-aged brains (# for *p* < 0.05). **(C)** Cycle number is unchanged in both middle-aged and old brains with repeated stimulation, but is significantly reduced at every stimulus in old brains compared to middle-aged brains (* for *p* < 0.05). **(D)** Onset latency in control experiments shows no quickening of onset latency in either middle-aged or old brains at follow up stimulus, but old brains are significantly slower at both stimuli (* for *p* < 0.05). **(E)** Cycle number in control experiments demonstrates no change with stimulus but significant decline with age (* for *p* < 0.05).

After we identified various parameters of behavioral locomotion that differentially undergo learning or behavioral loss with age, we then sought to identify neural correlates of these behavioral deficits. With this, we sought to determine if these changes were the result of CNS changes alone or whether they required the periphery. It is worth noting that the many studies of the basic electrophysiological properties of *Aplysia* neurons in elderly animals do not indicate that their neurons are any less able than those of younger animals to survive dissection and several hours of experimentation in artificial seawater (Kempsell and Fieber, 2015; Fieber et al., 2010; Peretz et al., 1982). In *Aplysia*, the locomotion efferent neurons are located in the pedal ganglia, which are a part of the central ganglia that make up the animal’s CNS (Figure 2A) (Fredman and Jahan-Parwar, 1980). Previous work has shown that a fictive motor program can be generated in an isolated brain via a stimulus to the tail nerve pedal nerve 9 (PdN9) (Bruno et al., 2015; Wang et al., 2023; Hill et al., 2020). To identify how aging specifically affects the *Aplysia* brain, we induced fictive locomotion in isolated *Aplysia* brains (middle-aged and old) via a stimulus to PdN9. As with the intact animals, in the experimental group (*n* = 6 for both middle-aged and old brains), we gave 5 stimuli 10 min apart, and in the control group (*n* = 6 for both middle-aged and old brains), we gave 2 stimuli 40 min apart (Figure 2A). We recorded the ensuing fictive motor program using voltage-sensitive dye imaging and extracted the true spiking activity of dozens of pedal efferent neurons per preparation to characterize the neural correlates of two locomotor parameters – onset latency (equivalent of head reach latency) and the number of locomotion cycles generated in 2 min.

In the experimental group, a RM two-way ANOVA did not identify significance in the interaction between repeated stimulation and age (*F*(4, 20) = 2.548, *p* = 0.07), but did identify significant effects on onset latency from repeated stimuli (*F*(4, 20) = 5.346, *p* = 0.004), and age (*F*(1, 5) = 18.68, *p* = 0.008) (Figure 2B). Multiple comparisons with Tukey’s post-hoc tests revealed a significant quickening in onset latency from stim 1 to stim 2 in middle-aged isolated brains (stim 1: 33.6 ± 0.7 s vs. stim 2: 20.4 ± 0.7 s, *p* = 0.03). However, differing from our behavioral results, this quickening faded by stim 3 (25.4 ± 1.4 s, *p* = 0.42), and remained unchanged by stim 5 (28.8 ± 2.4 s, *p* = 0.93), suggesting latency sensitization is more transient in the middle-aged isolated brain compared to the intact animal. In old isolated brains, by comparison, there was no sensitization of onset latency from stim 1 to stim 2 (stim 1: 39.4 ± 2.5 s vs. stim 2: 39.9 ± 3.2 s, *p* > 0.99), nor any significant change by stim 5 (46.6 ± 5.7 s, *p* = 0.58), once again demonstrating a loss of sensitization learning with aging – one not observed in the intact animal behavior. In the control group, a RM two-way ANOVA did not identify significance in the interaction between stimuli and age (*F*(1, 5) = 1.748, *p* = 0.24), and only identified a significant difference in onset latency from age (*F*(1, 5) = 35.73, *p* = 0.002), with no sensitization effect persisting across the 18 min stimulus gap (F(1, 5) = 4.636, *p* = 0.08) (Figure 2D). Multiple comparisons with Tukey’s tests in the control data showed no change in onset latency from stim 1 to stim 2 in middle-aged (stim 1: 28.5 ± 0.6 s vs. stim 2: 18.9 ± 1.2 s*, p* = 0.17) or old isolated brains (stim 1: 45.9 ± 2.4 s vs. stim 2: 43.3 ± 6.4 s, *p* = 0.91). However, there was a significant difference in onset latency between middle-aged and old brains at stim 1 (*p* = 0.02) and stim 2 (*p* = 0.04). Thus, while middle-aged isolated brains demonstrated onset latency sensitization, it did not persist across the 5 trials as it did in behavior, nor could it persist after a single trial for 40 min as observed in the intact animals. Old isolated brains were unable to sensitize at all, and middle-aged brains were less effective at staying sensitized than middle-aged animals, suggesting an earlier age-related decline in the CNS than observed in the intact animal.

Meanwhile, cycle number in the experimental group showed no effect from the interaction between stimulus and age (*F*(4, 20) = 0.5486, *p* = 0.70), and was unaffected by repeated stimulation in either group (F(4, 20) = 1.000, *p* = 0.43), but the absolute number of cycles was significantly reduced in the aged brains (*F*(1, 5) = 38.89, *p* = 0.002, mean of middle-aged: 7.9 cycles vs. mean of old: 3.7 cycles) (Figure 2C). This was also seen in the control group, where there was no significance in the interactions between stimulus and age (F(1, 5) = 2.426, *p* = 0.18) and no effect on cycle number by the first stimulus on the response to the second (*F* (1, 5) = 0.033, *p* = 0.86) but there was a significant effect from age (F(1, 5) = 61.49, *p* = 0.0005, mean of middle-aged: 9.4 cycles vs. mean of old: 3.8 cycles) (Figure 2E). Thus, while cycle number was unchanged within each age group with repeated stimulation, isolated brains from old animals generated far fewer cycles than those from middle-aged animals.

These results demonstrate that age-related behavioral and cognitive decline is also present in *Aplysia*’s CNS, and even reveal deficits unobserved in the intact animal behavior. This raises the possibility that early markers of aging are emerging in the isolated brain that are not yet observable in the whole animal.

### Repeated stimulation strengthens motor program response from middle-aged brains, weakens motor program response from elderly brains

After characterizing how age affected specific parameters of the fictive motor program (onset latency and cycling), we next sought to characterize whether aging affects the overall amount of firing from the pedal ganglion neurons during a fictive motor program. Our behavioral experiments identified a significant decline in galloping with age, which represents a more vigorous and intensive form of locomotion than crawling. We were interested in determining if aging also caused the locomotion network to become less vigorous by reducing overall population activity after a stimulus. To determine this, we calculated the average spike frequency over time in each VSD experiment described in the previous section (*n* = 6 for both middle-aged and old brains), to determine if overall neuronal activity changed due to age or successive trials within the first 2 min after a stimulus.

**Figure 3:**
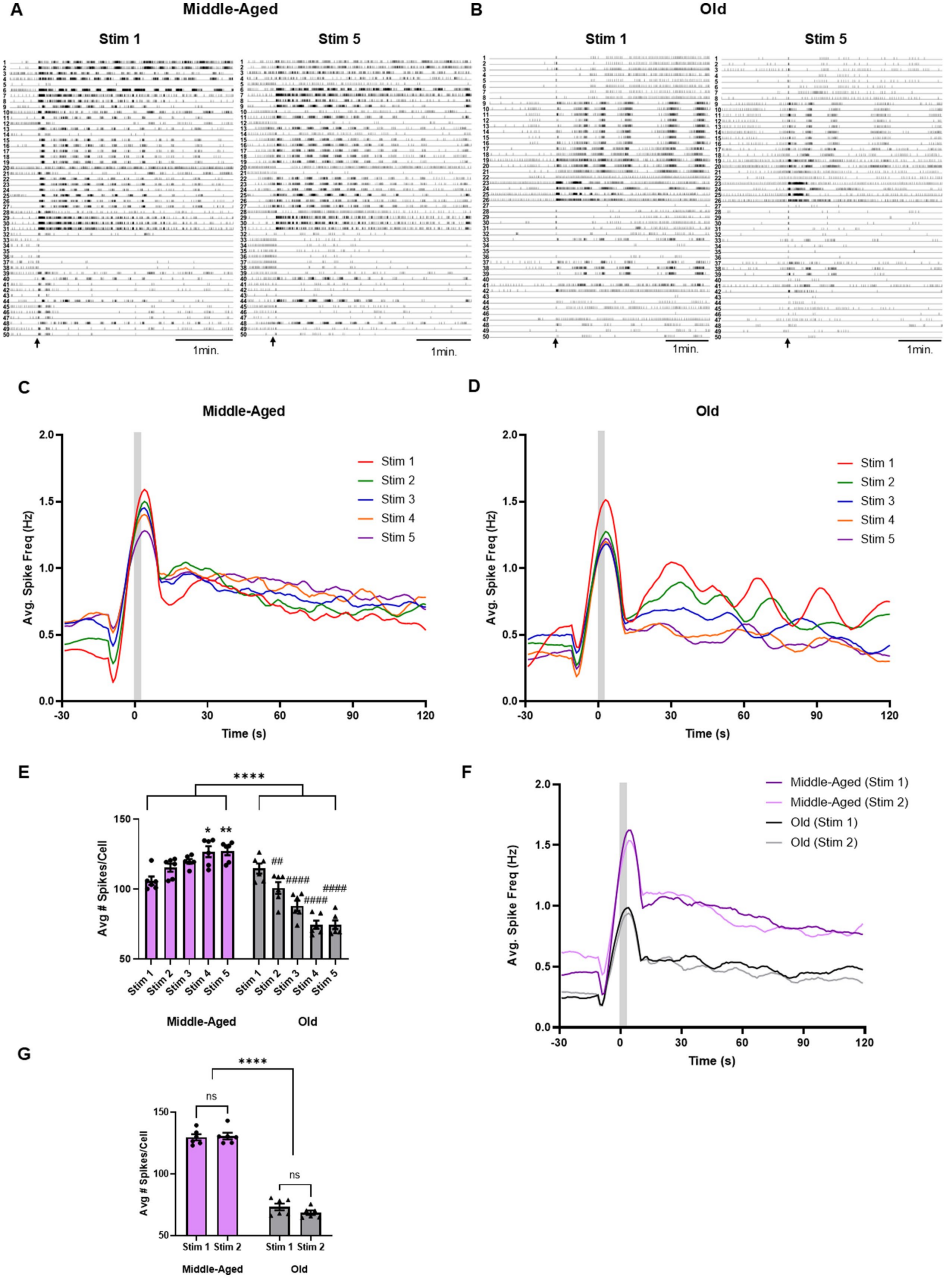
Aging reduces neural activity following a stimulus. **(A)** Example of spike rasters generated from VSD imaging in the same middle-aged isolated brain at stim 1 (left) and stim 5 (right). First 50 neurons identified with ICA displayed. Each numbered neuron in stim 1 is the same neuron in stim 5. Stimulus indicated with black arrow. **(B)** Same type of raster demonstrated in **(A)**, but from an old brain. **(C)** Averaged spike frequency graphs for all recordings at each experimental stimulus in middle-aged brains. Rolling 10 s average spike frequency plotted. **(D)** Same type of graph as **(C)**, but with old brains under experimental condition. **(E)** Average spikes/cell significantly increased with follow-up stimuli in middle-aged isolated brains (* for *p* < 0.05, ** for *p* < 0.01). In comparison, average spikes/cell significantly decreased with follow-up stimuli in old isolated brains (## for *p* < 0.01, #### for *p* < 0.0001). *N* = 6 recordings for both middle-aged and old brains. **(F)** Average spike frequency graphs for control recordings in middle-aged (purple) and old (grey) isolated brains. Rolling 10-s average spike frequency plotted. **(G)** Average spikes/cell did not change in control condition within either middle-aged or old brains, but was significantly reduced in old brains (**** for *p* < 0.0001). *N* = 6 recordings for both middle-aged and old brains.

We compared changes in neuronal activity between our middle-aged and old groups (Figures 3A,B) by determining the average spike frequency for each VSD preparation and comparing changes in the average number of spikes per cell across the entire recording (Figures 3C,D). In our experimental group, a two-way RM ANOVA found significant interactions between stimulation and age (*F*(2.321, 23.21) = 120.5, *p* < 0.0001), as well as from repeated stimulation on average neuronal activity over time (F(2.321, 23.21) = 5.582, *p* < 0.0001) and age (*F*(1, 10) = 48.64, *p* < 0.0001) (Figure 3E). Tukey’s tests found significant increases in the average number of spikes per neuron in the middle-aged group from stim 1 to stim 4 (stim 1: 106.0 ± 3.0 vs. stim 4: 126.7 ± 3.9, *p* = 0.01), and stim 5 (127.1 ± 2.9, *p* = 0.006). This indicated that the middle-aged brains were producing progressively stronger fictive locomotion with each successive stimulus, an example of sensitization. In our old experimental group, by comparison, we observed a steady decrease in activity with repeated stimulus trials. Tukey’s post-hoc tests identified significant decreases in the average # of spikes/cell in the old group from stim 1 to stim 2 (stim 1: 114.9 ± 3.6 vs. stim 2: 100 ± 4.2, *p* = 0.004), stim 3 (88 ± 3.7, *p* < 0.0001), stim 4 (74.4 ± 2.9, *p* < 0.0001), and stim 5 (74.7 ± 3.1, *p* < 0.0001). Thus, old brains were producing progressively weaker fictive locomotion with successive stimuli, an example of habituation, even as the number of cycles produced remains the same. In our control group (Figure 3F), a two-way ANOVA revealed no significance in the interactions between stimulation and age (*F*(1, 10) = 3.556, *p* = 0.09), and no effect from stimulation (*F*(1, 10) = 1.455, *p* = 0.25), only an effect from age (F(1, 10) = 363.2, *p* < 0.0001, mean of middle-aged: 130.5 vs. mean of old: 70.6), indicating that old brains in general produce weaker fictive locomotion, a clear effect of aging (Figure 3G). These results demonstrate that middle-aged brains produce more intensive locomotion that sensitizes with repeated stimulation. In old brains, however, the network starts off weaker and habituates with repeated stimulation.

The network activity changes in both middle-aged and old brains, but are these changes occurring in a consistent fashion across the repeated motor programs in the two age groups? To answer this, we tested if a single curve could fit all the trials in middle-aged or old recordings. We found that a single two-phase decay curve could fit all the spike frequency data in the middle-aged experimental group (*r*^2^ = 0.38). Thus, repeated stimulation made the middle-aged brains more vigorous by increasing their spike frequency in a consistent fashion across the whole length of the fictive motor program. In old brains, however, we were unsuccessful in fitting a singular non-linear curve to the set of experimental graphs post-stim. We found that separate one-phase decay graphs best fit the curves for stim 1 (*r*^2^ = 0.29) and stim 2 (*r*^2^ = 0.26), while separate two-phase decay graphs best fit for stim 3 (*r*^2^ = 0.40), stim 4 (*r*^2^ = 0.52), and stim 5 (*r*^2^ = 0.55). Therefore, the old brains are not simply becoming less active across the board, but are also gradually shifting activity in a way that makes them more erratic and less comparable from trial to trial. This suggest further deficits at a network level in aged brains beyond reduced firing rate.

These results demonstrated that in middle-aged *Aplysia*, repeated stimulation produced a sensitization-driven increase in network activity across trials, consistent with prior published findings of increased distance travelled with successive stimulation (Stopfer and Carew, 1988). With age, the effect is flipped – repeated stimulation produces habituation of network activity across trials. This reduced response from the aged network is likely the result of CNS aging. This reduction in output from aged brains to a stimulus may explain the decline in galloping response observed in aged animals, as the reduced activity takes the form of weaker firing during the bursts that produce the locomotion cycles.

### Aging also reduces the activity and rhythmicity of resting efferent neurons

The reduced average neuronal activity in aged brains during motor programs made us consider other properties of the efferent neurons that are also declining with age. Aging reduces neuromodulator expression in *Aplysia* (Flinn et al., 1997) and alters sensory and motor neuron excitability in a different *Aplysia* behavior, the tail withdrawal reflex (Kempsell and Fieber, 2014; Kempsell and Fieber, 2015), which could result in a general reduction in pedal efferent neuron activity, before and after a stimulus. To determine if aging reduced the resting activity of pedal neurons, we prepared *Aplysia* isolated brains for VSD recording as before, but then only recorded spontaneous activity in the pedal ganglia for 5 min in middle-aged (*n* = 7) and old (*n* = 7) isolated brains, with no stimulus-elicited motor program during or prior to the recording.

**Figure 4:**
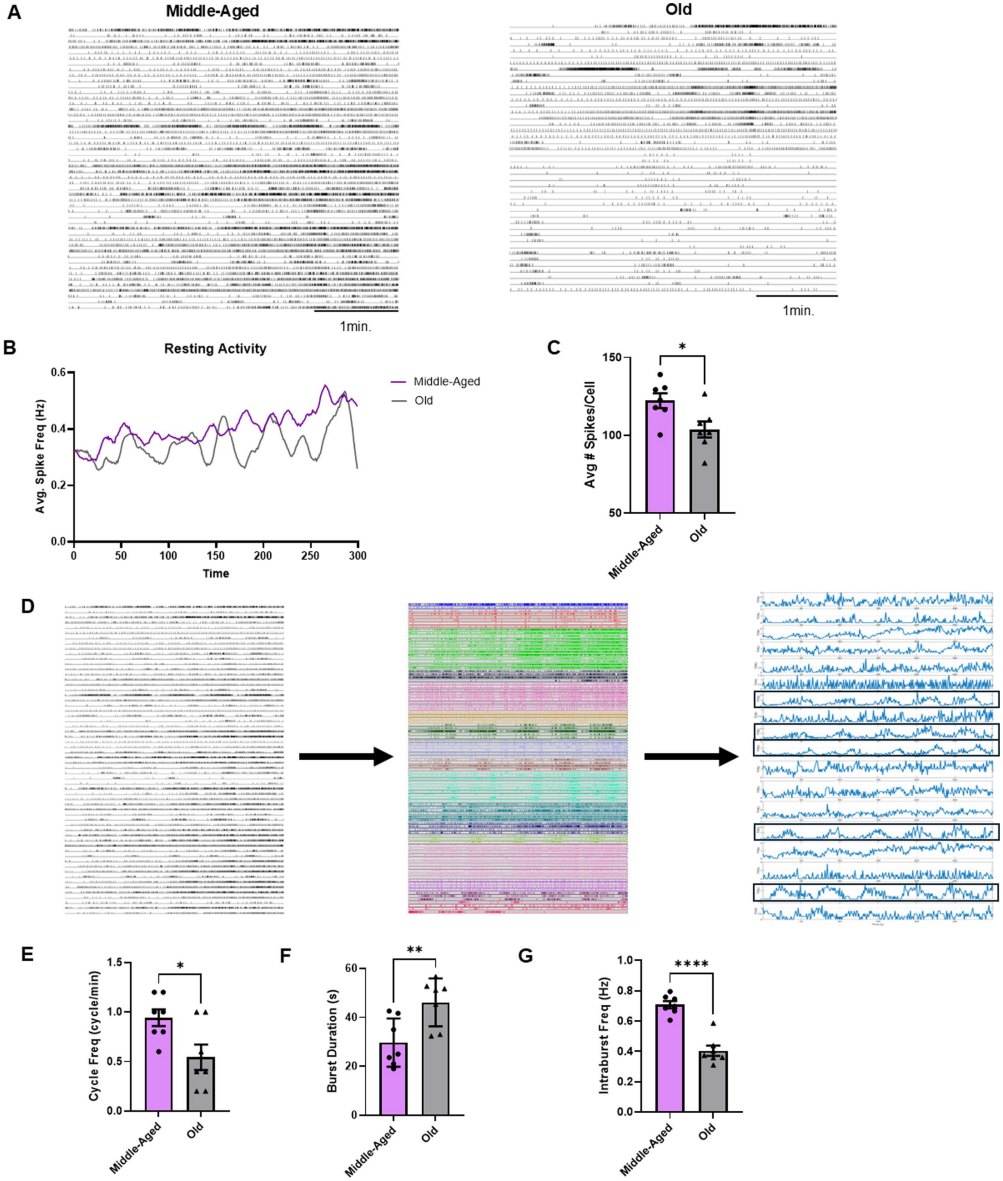
Aging reduces resting state activity in the pedal ganglion. **(A)** Examples of spike rasters from VSD imaging in middle-aged and old pedal ganglia after 1 h of rest. Middle-aged pedal neurons demonstrate noticeably more activity at rest compared to old pedal neurons. **(B)** Average spike frequency graphs of all recordings of middle-aged (*n* = 7) and old (*n* = 7) isolated brains at rest. Rolling 10 s average plotted. **(C)** Average spikes/cell of resting state activity shows a significant decrease from middle-aged to old (* for *p* < 0.05). **(D)** Workflow for characterizing resting rhythm, as seen in spike trains on the left. Spike trains of neurons (left) underwent unsupervised consensus clustering, producing statistically correlated functional clusters (middle). Average spike frequency is determined for each functional cluster (right) and power spectral density was used to identify which clusters are rhythmic (noted with black boxes) for calculation of parameters shown in **(E–G)**. **(E)** Cycle frequency of resting rhythm decreases significantly in old brains (* for *p* < 0.05). **(F)** Burst duration of resting rhythm bursts increases significantly in old brains (** for *p* < 0.01). **(G)** Intraburst frequency decreases significantly in old brains (**** for *p* < 0.0001).

We first compared average neuronal activity during these spontaneous activity recordings between middle-aged and old brains as in the previous section, by comparing the average number of spikes per cell during a VSD recording (Figures 4A,B). We found that the # of spikes/cell for middle-aged brain spontaneous activity was significantly greater than that of old brains (*t*-test, middle-aged: 121.3 ± 4.7 vs. old: 103.7 ± 5.5, *p* = 0.02) (Figure 4C). This reduction of spontaneous activity at rest indicates a generalized effect of aging on the activity of the efferent neurons.

**Figure 5:**
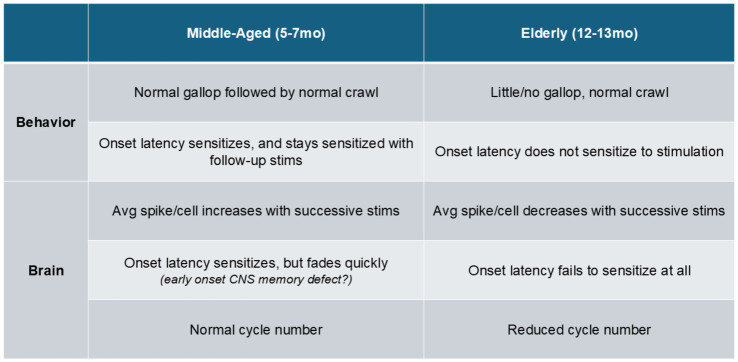
Summary of how aging affects *Aplysia* locomotion. Aging produces cognitive deficits in both intact animal behavior and isolated brain activity. Older *Aplysia* lose the ability to shorten initial head reach latency, and show a significant reduction in galloping. In their brains, we observed reduced neural activity at rest and after successive stimuli. Notably, middle-aged brains also demonstrate an inability to have onset latency sensitization persist, which could be an early sign of age-related decline that is currently masked in the intact behavior.

Our VSD imaging detected the presence of a slow bursting rhythm in the resting activity (Figure 4D). This is consistent with a recent study in intact behaving middle-aged *Aplysia* that also identified a slow spontaneous rhythm of activity at rest in pedal nerve 10, which is often used to record the locomotion motor program (Wang et al., 2023). To determine if this resting rhythm was diminished in aged preparations, we first developed a methodology for discriminating rhythmic and non-rhythmic neurons. We used a previously published unsupervised clustering algorithm (Bruno et al., 2015) to organize neurons into identified functional clusters and then used power spectral density to determine if these functional clusters were rhythmic (Figure 4D). If a cluster was rhythmic, the cycle frequency, burst duration, and intraburst frequency for the whole cluster was then calculated and averaged for each recording, allowing us to quantify this resting rhythm in middle-aged neurons and see if it was affected by aging. Unpaired t-tests identified significant decreases in cycle frequency (middle-aged: 0.94 ± 0.08 cycles/min vs. old: 0.54 ± 0.13 cycles/min, *p* = 0.02), burst duration (middle-aged: 29.7 ± 3.7 s vs. old: 46.2 ± 3.7 s, *p* = 0.009) and intraburst frequency (middle-aged: 0.71 ± 0.02 Hz vs. old: 0.40 ± 0.03 Hz, *p* < 0.0001) in old brains compared to middle-aged brains (Figures 4E–G). Thus, this slow resting rhythm observed in middle-aged *Aplysia* pedal neurons is significantly weaker in older brains, representing another feature of aging in the neurons that participate in the motor network (see Figure 5).

These results demonstrated a significant weakening of resting activity in old *Aplysia* pedal neurons, both in overall activity and with respect to a slow spontaneous bursting rhythm that may or may not correspond to slow crawling. This broader reduction in activity in pedal neurons at rest suggests that aging is broadly affecting the constituent members of the locomotion network. The reduced activity post-stimulus in aged brains is therefore likely produced by a combination of age-related decline in both the CPG and the pedal efferent neurons.

## Discussion

As aging produces complex, heterogeneous declines in behavior and memory formation, simpler model systems present the opportunity to more easily trace age-related behavior and cognitive losses to their underlying neural causes. In this study, we show for the first time how aging affects motor function and memory formation within *Aplysia* escape locomotion (see Figure 5 for summary of findings). These findings lay the foundation for future studies to investigate how to mitigate and rescue age-related decline in this behavior, which may reveal insights about aging that are generally relevant across species.

### Behavioral and cognitive age-related decline emerge within specific components of *Aplysia*’s escape behavior

We began this study with an effort to characterize how non-associative learning in *Aplysia*’s escape behavior is changed by aging. However, the two main forms of non-associative learning, sensitization and habituation, can be encoded differently into the same network depending on stimulus interval and intensity (Groves and Thompson, 1970). Work in *C. elegans*’ reversal response has demonstrated varied responses to habituation training depending on inter-stimulus interval (Rankin et al., 2009). In the nudibranch mollusk *Tritonia diomedea*’s escape response, the same behavioral parameters can sensitize or habituate differently depending on stimulus intensity and interval (Frost et al., 1996). In the same set of animals, one parameter of *Tritonia’s* escape behavior (onset latency) can even remain sensitized as another component of the escape behavior (cycle number) habituates (Mongeluzi and Frost, 2000). In our experiments here, we studied three different parameters of the *Aplysia* escape behavior: head reach onset latency, cycle number, and gallop share of all cycles. The only parameter of these in middle-aged *Aplysia* that underwent either sensitization or habituation was onset latency, which sensitized after an initial stimulus and remained sensitized with repeated stimuli. It is possible that a different stimulus protocol could produce different results, and further work is needed to identify other behavioral parameters of this behavior that may also be affected by learning. In our elderly animals, by comparison, we observed a loss of onset latency sensitization, the first identified site of cognitive aging within this behavior. While cycling remained robust in these aged animals, their galloping response was largely abolished. Thus, within this behavior, one parameter underwent cognitive aging (latency sensitization), another underwent behavioral aging (gallop share), and another is unaffected by aging (cycle number).

Distinct impacts from aging on various components of the same network are consistent with a larger body of work demonstrating the heterogeneity of aging in behavioral circuits, even in simple invertebrates. For instance, while aging causes impaired response intensity and faster habituation in *Aplysia*’s gill-siphon withdrawal reflex, it has no effect on gill respiratory pumping (Peretz and Srivatsan, 1992). The authors hypothesize this difference in aging effects is the result of either increased usage of the respiratory pumping pathway in aged animals, or the relative necessity of respiratory pumping compared to gill/siphon withdrawal. As crawling locomotion is a necessary behavior that *Aplysia* use to find food, mates, and areas of safety (Wang et al., 2023), this may explain why crawling remains robust in aged animals.

These differences in behavioral aging may also be the result of different neurons within the network aging differently. In the freshwater mollusk *Lymnaea stagnalis*, aging causes feeding behavioral deficits through changes to specific synapses in the network (Yeoman et al., 2008), even different synapses made by the same presynaptic neuron (Klaassen et al., 1998). In *Aplysia*, two different cholinergic neurons of the gill-siphon withdrawal reflex show distinct age-related changes to their genomes, including to genes responsible for neuronal function and synaptic plasticity (Moroz and Kohn, 2010). If the neurons responsible for the various elements of the locomotion behavior are aging differently, this could explain why some parameters are affected by aging when others are age-invariant.

While aged *Aplysia* did not undergo onset latency sensitization from repeated stimulation, we found it interesting that their onset latency after the first stimulus was already quick, compared to the middle-aged *Aplysia* which started off more slowly (Figure 1B). In our isolated brain experiments, those from old animals also did not quicken their latency with repeated stimulation, but started off relatively slow compared to middle-aged brains and remained slow even with repeated stimulation (Figure 2B). As a result, we would not suggest the behavioral results indicate that older animals are inherently quicker at initiating locomotion. Instead, we suggest that in our behavioral experiments, middle-aged *Aplysia* adapted to the new context of the testing arena and were in a state of rest during delivery of the first stimulus, while the old *Aplysia* were not. *Aplysia* are known to sleep (Vorster et al., 2014), and sleep deprivation or disruption has been shown to interfere with learning in other *Aplysia* behaviors (Krishnan et al., 2016; Krishnan et al., 2016). Age-related sleep disruption has been demonstrated in many species (Zhong et al., 2019) and has been demonstrated to contribute to cognitive loss in elderly humans (Zimmerman et al., 2012). One possibility is thus that the ability of our aged animals to rest is disrupted by the change in context when they are moved into the testing area, which behaviorally manifests as a quickened latency in intact animals. Deafferentation, however, eliminates this confound, and reveals the extent of CNS cognitive and behavioral aging via a slower latency in isolated brains that are unable to quicken as a result of repeated stimulation.

### Differences in behavioral and cognitive aging between isolated brains and intact animals

One central advantage of utilizing marine invertebrates for studying the neural basis of behavior and learning has been that their motor programs can be reliably reproduced in isolated brain or semi-intact preparations permitting behavior. The gastric/pyloric rhythm in crustaceans (Marder and Bucher, 2007), the escape swim of *Tritonia* and *Pleurobranchaea* (Katz and Sakurai, 2017), and the escape response of medicinal leech (Kristan et al., 2005) are all examples of simple behaviors in invertebrates that have been robustly interrogated in isolated preparations precisely because the fictive network activity in their neurons closely mirrors the real behavior of the intact animal. *Aplysia* has made numerous contributions to this field of work as well – *Aplysia*’s gill-siphon withdrawal reflex (Carew and Kandel, 1973), tail withdrawal reflex (Walters et al., 1983), feeding motor program (Kupfermann and Weiss, 1981), and escape locomotion (Jahan-Parwar and Fredman, 1979) have all been fictively generated in isolated brain preparations with time courses and activity patterns that are consistent with what is observed in the intact animal, supporting decades of work exploring the neural basis of these behaviors as well as different forms of learning within them.

We sought to determine whether the behavioral and cognitive aging we observed in our intact animal experiments persisted in isolated brains, as aging is also known to affect other body systems such as the muscles (López-Otín et al., 2013). We observed cognitive aging in one parameter of isolated brains – onset latency of the locomotion motor program could sensitize in brains from middle-aged but not old animals. It is worth noting that onset latency sensitization in middle-aged brains was more transient than in middle-aged animals (Figure 2B vs. Figure 1B), and persists for longer in intact middle-aged animals than in their isolated brains (Figure 1E vs. Figure 2D), evidence that the learning fades faster in isolated middle-aged brains than in the intact animal. Additionally, while we also observed no change in cycling with repeated stimulation within either age group in either intact animals or isolated brains, we did observe an overall reduction of cycling in all trials in elderly brains compared to middle-aged brains (Figures 2C,E). These observations of CNS-specific deficits may represent network-level signatures of age-related dysfunction that have yet to manifest in the intact behavior. This presents an exciting opportunity for future experimentation and study into when chronologically these CNS deficits emerge, and if these CNS deficits represent early predictors for future motor and cognitive decline in whole animals.

It is additionally possible that the discrepancy in results between isolated brain and whole animal experiments is due to contributions from the peripheral nervous system (PNS). The role of the PNS in regulating behavior and learning has been previously explored in marine invertebrates. The PNS has been demonstrated to make critical contributions to the development of and persistence of habituation in *Aplysia*’s gill-siphon withdrawal reflex (Goldberg and Lukowiak, 1982; Goldberg and Lukowiak, 1984; Leonard and Edstrom, 2004; Lukowiak and Peretz, 1977). The nudibranch mollusk *Berghia stephanieiae* utilizes both CNS and PNS processes to produce a defensive posturing behavior known as bristling, and the PNS is able to override the CNS under certain circumstances (Brown et al., 2024). The interactions between octopus brain and arms have led to a model of a distributed network where the CNS and PNS work synergistically to generate behavior, a framework for embodied intelligence (Hochner, 2012; Sumbre et al., 2001; Levy and Hochner, 2017; Hochner et al., 2023). It is possible that the PNS may play a role in both normal behavioral function and learning in *Aplysia* escape locomotion, and may have a role in compensating for age-related declines in brain function.

Aging has been shown in both invertebrates and vertebrates to affect the interactions between the CNS and PNS. In *Lymnaea,* semi-intact lip-CNS preparations of the feeding network have identified age-related decreases in CNS output, but not in PNS input – demonstrating that aging specifically affected central but not peripheral processing (Arundell et al., 2006). Aging has also been shown to affect the level of central versus peripheral control of habituation learning in *Aplysia*’s gill-siphon withdrawal reflex (Peretz and Lukowiak, 1975). In both mammalian animal models and in human studies, there is growing interest in the role of the autonomic nervous system (ANS) in typical brain aging and utilizing ANS variability to predict neurodegenerative diseases (Lin and Heffner, 2023; Giunta et al., 2024). Future experiments utilizing semi-intact preparations of the *Aplysia* escape behavior may have the potential to elucidate how aging shifts the contributions of the PNS and CNS in order to maintain the production of regular behavior in elderly animals.

### Aging diminishes and disrupts network activity, before and after a stimulus

Simple marine invertebrates possess large neurons with easily accessible somata that make them very amenable to large-scale activity imaging with voltage-sensitive dyes (Hill et al., 2014). Technical advances have enabled the ability to capture the voltage activity of dozens of neurons with single-cell, single-spike resolution (Hill et al., 2010). VSD imaging has facilitated investigations into how neural networks produce behavior in multiple invertebrate species, including *Tritonia* (Hill et al., 2012), *Berghia* (Hill et al., 2020), crabs (Preuss and Stein, 2013), and medicinal leeches (Tomina and Wagenaar, 2017). VSD imaging has also been used to reveal novel network-level properties of learning within the *Tritonia* escape swim (Hill et al., 2015). In *Aplysia,* VSD imaging has facilitated investigations into the networks that produce the gill-siphon withdrawal reflex (Zecevic et al., 1989), feeding (Costa et al., 2022), and escape locomotion (Bruno et al., 2015; Bruno et al., 2017). We sought to apply this technique to study how aging affects the locomotion network’s ability to reorganize itself during non-associative learning.

In middle-aged isolated brains we observed a steady increase in the average spikes/cell of the fictive motor program with each successive stimulus (Figure 3E). Since we did not observe an increase in the cycle number in these recordings, this suggests that each cycle performed was more intense, which seems likely to translate to an increase in distance travelled over time. This would be consistent with a prior study of the development of *Aplysia* escape locomotion sensitization in juvenile animals, which found an increase in distance travelled after a sensitizing stimulus (Stopfer and Carew, 1988). In our VSD recordings from old brains, by comparison, we observed a steady decrease in average spikes/cell with each successive stimulus. These divergent results suggest that aging is not simply causing a reduction in specific parameters of the motor program, but may also be altering how the overall network processes repeated stimuli and correspondingly generates motor programs. We would also expect this to result in reduced distance travelled, but further work is needed to confirm whether this is the case.

How does repeated stimulation produce these increases in activity in youth, and decreases in activity with old age? One potential explanation is serotonin modulation of both the CPG and the efferent neurons. Prior work has demonstrated that one of the primary modulators of *Aplysia’s* locomotion behavior is serotonin (Mackey and Carew, 1983; Marinesco et al., 2004), and many of the pedal efferent neurons possess 5-HT_1A_ and/or _2A_ receptors (Barbas et al., 2003). Injection of serotonin precursor (5-HTP) is also sufficient to elicit spontaneous locomotion in middle-aged animals (Marinesco et al., 2004). Sensitization learning in different *Aplysia* behaviors utilizes serotonin modulation to modify intrinsic properties, synaptic strength, neurotransmitter release, and tonic firing (Byrne et al., 1991; Frost et al., 1988). However, dopamine is also a prominent neuromodulator of this motor program that is responsible for transitioning the network from gallop to crawl, and many pedal neurons also possess D_1–4_ receptors (Flinn et al., 2001; Chandhoke et al., 2001). We therefore speculate that repeated activation of this motor network induces multiple modulatory changes via serotonin and dopamine to the CPG and efferent neurons that ultimately result in a more vigorous escape motor program. This argument is supported by our demonstration of reduced vigor in the aged network with repeated stimulation. Aged *Aplysia* are known to have lower serotonin expression levels and reduced expression of dopamine receptors in pedal neurons (Flinn et al., 1997; Chandhoke et al., 2001), so it is possible that this reduced response vigor in aged brains is the result of decreased or disrupted neuromodulation. It is also possible that aging causes a reduction in CPG neurotransmitter release onto the efferent neurons. Habituation in *Aplysia*’s gill-siphon withdrawal reflex has been demonstrated to be produced in part by reduced transmitter release (Castellucci and Kandel, 1974). While the middle-aged escape response does not habituate in our protocol, it is possible that habituation develops with age due to the reduction in modulator expression. It is also possible that if we extended the training beyond 5 trials, habituation might be observed.

We also observed differences in how network activity post-stimulus changed based on age. While middle-aged brains increased their output with each successive stimulus, they did so in a consistent fashion over time, such that a single curve was sufficient to fit every post-stimulus activity graph. We hypothesize that this is because the pedal efferent neurons have become more intrinsically excitable as a population, such that the same amount of CPG output produces a consistently stronger efferent response. Foundational work in *Aplysia*’s gill-siphon withdrawal reflex has identified such changes in sensory neurons during sensitization learning (Walters et al., 1983). Learning induced changes in excitability have been explored in other invertebrates – the mollusk *Hermissenda crassicornis* alters the excitability of visual-vestibular photoreceptors during associative learning (Alkon et al., 1982; Alkon et al., 1986). Altering the excitability of a network during learning has also been observed in various mammalian brain regions, and as such represents a conserved mechanism for learning-induced network plasticity (Josselyn and Frankland, 2018). Further work is needed to test this hypothesis, and to determine what cellular mechanisms may be potentially involved.

Aging has been shown to reduce intrinsic excitability and synaptic input to a critical motor neuron in the *Aplysia* gill-siphon withdrawal reflex (Rattan and Peretz, 1981; Peretz et al., 1982), and decrease intrinsic excitability and synaptic strength in mammalian circuits (Chen et al., 2023; Detoledo-Morrell et al., 1988). As a result, we were not surprised to see that the aged network responded less intensely to an initial stimulus and became less intense with repeated stimulation. However, repeated stimulation reduced neural activity in an inconsistent fashion – unlike with middle-aged preparations, a different decay curve was needed to fit each post-stimulus graph. This suggests that in addition to potential reductions in neurotransmitter tone and reduced excitability of aged efferent neurons, there are other disruptions to the aged network occurring with repeated stimulation that disrupted the activity post-stimulus. This may suggest that aging is also interfering with network features not readily evident by simply looking at spiking activity, a topic left for future studies.

In addition to changes in neuronal activity, aging is known to affect many different biological and physiological functions (López-Otín et al., 2013; López-Otín et al., 2023). In *Aplysia*, aging has been shown to disrupt second messenger signaling and cellular metabolism in tail sensory neurons (Kron and Fieber, 2022; Badal et al., 2024; Greer et al., 2018), likely contributing to behavioral and cognitive aging in its tail withdrawal reflex. We therefore utilized our VSD imaging technique to determine if aging altered the activity of locomotion pedal neurons at rest. This would allow us to indirectly assess the degree to which reduced fictive locomotor activity is the result of specific deficits in the CPG versus deficits across the efferent neurons as well. We did observe a decrease in network activity at rest in old pedal ganglia compared to middle-aged pedal ganglia (Figure 4A). This seemed to be driven by a significant weakening of the slow oscillatory activity we observed in many middle-aged pedal neurons at rest. This slow oscillation, which we term the “resting rhythm,” has been observed in nerve recordings in middle-aged intact animals (Wang et al., 2023), and is present in many neurons that burst rhythmically during the motor program. While we do not currently know the function or source of this resting rhythm, its weakened state in aged brains suggests further deterioration within the CNS that may affect the fictive locomotion motor program.

What is the role of this resting rhythm in the pedal neurons? We hypothesize it represents a slow background readiness state for the locomotion behavior that is disrupted in aging. This disruption could be driven by a combination of reduced upstream input as well as degraded intrinsic properties of pedal neurons. In humans, fMRI studies have identified a series of brain regions active at rest that are collectively referred to as the “default mode network” (DMN) (Sambataro et al., 2010). While the role of the DMN remains a topic of investigation, arguments have been made about its role in cognition (Smallwood et al., 2021). In older humans experiencing typical brain aging, the DMN has been shown to be reduced in several critical regions involved in learning, including the hippocampus (Mevel et al., 2011). The DMN is also further dysregulated in older patients with neurodegenerative diseases like Alzheimer’s disease (Rombouts et al., 2005). However, the indirect nature of human neuroimaging studies has limited the ability to directly study or manipulate the DMN. Future work in attempting to rescue age-related behavioral and cognitive decline in *Aplysia* could consider measuring changes in this resting rhythm to determine if the effects are localized to the fictive motor program or are broadly and generally improving the aged brain, both during and outside behavior.

While this study focused on how aging affects behavioral function and learning in an escape locomotion context, it may have implications for work in non-motor related conditions as well. Locomotion tends to be stereotyped, rhythmic, and fundamental to survival, both under escape and non-escape conditions. If a critical survival behavior, which is required to be produced into old age, has undergone deterioration, what might happen to less frequently activated circuits within the brain? There is reason to suspect that such essential and regularly activated circuits are protected into old age. As mentioned previously, a prior study in elderly *Aplysia* looked at two behaviors that share peripheral organs and have overlapping neurons in the abdominal ganglion – respiratory pumping, which is regularly produced and is critical to survival, and the gill-siphon withdrawal reflex, which occurs less frequently in the wild (Peretz and Srivatsan, 1992). The authors found old age caused deterioration in the withdrawal reflex, but not in respiratory pumping, and suggest that the latter’s regular activation and essentialness for survival protected it into old age. We speculate that the deficits and the potential silent signatures of age-related decline would be even worse in neural networks that are not as regularly activated and essential as locomotion.

Studying how aging produces deficits in motor behavior requires a multi-scale experimental approach, integrating findings from the molecular and genetic level to changes in individual neurons to network-level properties. While this study advances our understanding of how aging affects *Aplysia*’s locomotion at both the behavioral and neuronal activity level, and represents the first VSD study we know of in aged *Aplysia*, we still require further experimentation to understand how aging is affecting the transcriptome, intrinsic membrane properties, and lower-dimensional network properties of this behavioral network. While we anticipate some of these age-related changes will be specific to *Aplysia*, we believe certain components of this will be generally applicable. Prior work on *Aplysia*’s locomotion network has shown that the high-dimensional neuronal population activity emerges from a low-dimensional spiral attractor (Bruno et al., 2017), and analysis of a different population of *Aplysia* neurons has revealed shared transcriptomic and genetic markers of age-related neurodegeneration with mammals (Kron and Fieber, 2022; Badal et al., 2024). While the *Aplysia* escape locomotion network is a relatively stereotyped and consistent motor program in an invertebrate, we believe further investigation into how aging affects this behavior at the lower-dimensional population code level will help uncover more generally conserved mechanisms of aging.

### *Aplysia* escape locomotion as a novel paradigm for exploring age-related decline of rhythmic behavior

Behavioral and cognitive aging have been observed across various species and continue to be of strong clinical relevance to humans. To effectively study how aging develops within the brain and develop interventions to mitigate and rescue age-related decline, however, there is a benefit to utilizing systems with simple nervous systems to more directly draw comparisons from brain activity to behavior. *Aplysia* escape locomotion is well positioned as a paradigm for continued exploration into behavioral and cognitive aging, as it is a multi-phase rhythmic network with complex modulation that is highly amenable to network-level analyses of neural activity. We have demonstrated here that aging affects both the escape behavior and the neural network that produces it in heterogeneous ways. These findings pave the way for future work investigating how network function declines with age and what types of interventions can successfully prevent or rescue said decline.

## Data Availability

The raw data supporting the conclusions of this article will be made available by the authors, without undue reservation.
